# Fatal Errors

**Published:** 1881-05

**Authors:** Ad. Lippe

**Affiliations:** Philadelphia


### FATAL ERRORS.

BY AD. LIPPE, M.D., PHILADELPHIA.

It is a fatal error to contend that the posological question divides homœopathists into "High Potency" and "Low Potency" parties. This fatal error is designedly made by *The Observer* in its issue of February, 1881.

It is an indisputable fact that there has existed, for a long time, a difference or opinion as to our posology; but that question never, at any time, formed the line of demarcation between homœopaths and eclectics. It was and is a historical fact that the men who followed the strict inductive method of Hahnemann cured the sick by means of infinitesimal doses, while the others, who claimed the right to be governed by their own individual opinions, irrespective of Hahnemann's strict inductive method, resorted to massive doses and finally, to palliatives, in their vain efforts to cure the sick. All the time they facetiously claimed superior successes, which they never showed or demonstrated, and also claimed to be as good homœopathists as any. But now, it is also a historical fact that the posological question no longer exists, at all. Prof. Gustav Jaeger, in his communications on Neuralanalysis, which were published in the January (1881) number of *The Organon*, says: "The numerically shown, generally very considerable, increase of the physiological action of a medical substance by potentization elevates homœopathy, by one stroke, to the rank of an exact, physiologically based method of cure, undeniably of equal birthright with allopathy. In consideration of the easy access which neuralanalysis offers for the formation of a verdict, it will hereafter be impossible for our Universities to continue their systematic and persistent persecution of the homœopathic school." Not only this, but under Sections five and six, are mentioned subjective effects caused by the inhalation of high potencies, for a quarter of an hour; "showing such strong results as far exceeded my expectations." Among all the men of learning, among the allopathic physicians who, *ex cathedra*, deny the effects of homœopathic dilutions, there is not to be found one who has made the experiment, as becomes an expert; if he had done so he would at least have halted, and been surprised at his results. The defenders of allopathy will herewith suffer not only a scientific, but also a moral, defeat; as the heaviest charge one can bring against an expert is that he rendered a verdict without taking the least pains to institute an examination becoming an expert. And especially a verdict of such gravity that, with it, if it were just, numerous persons of the educated and professional class were to be stamped as swindlers, cheats or as cheated.

Be it remembered that Prof. Jaeger is a professor of natural sciences in an allopathic school. Be it remembered also that it is a fatal error to claim that any progress in science ever modified or annulled any of the strict inductive methods of Hahnemann; to the contrary, all and every advance in science develops and proves the correctness of our healing art. Here we have seen a scientist, *by one stroke*, settle the burning posological question and all disputes growing out of it; this scientist proves by ocular demonstration that the sick-making power of drugs is increased manifold by potentization! Where are the heroes of Milwaukee memory? Where are the diligent microscopists? Why wiped out *by this one stroke!* And where is the sage and philosopher, Hahnemann? Why, endorsed by every new discovery made in any of the sciences.

